# Blood gases, biochemistry and haematology of Galápagos hawksbill turtles (*Eretmochelys imbricata*)

**DOI:** 10.1093/conphys/cox028

**Published:** 2017-05-10

**Authors:** Juan Pablo Muñoz-Pérez, Gregory A. Lewbart, Maximilian Hirschfeld, Daniela Alarcón-Ruales, Judith Denkinger, Jason Guillermo Castañeda, Juan García, Kenneth J. Lohmann

**Affiliations:** 1 Galapagos Science Center, University San Francisco de Quito, Puerto Baquerizo Moreno, Galapagos, Ecuador; 2 College of Veterinary Medicine, North Carolina State University, 1060 William Moore Drive, Raleigh, NC 27601, USA; 3 Galapagos National Park Service, San Cristóbal, Galapagos, Ecuador; 4 Department of Biology, University of North Carolina, Coker Hall, Chapel Hill, NC 27599, NC, USA

**Keywords:** Biochemistry, blood gas, chelonians, *Eretmochelys imbricata*, Hawksbill turtle, health, haematology, reptiles

## Abstract

The hawksbill turtle, *Eretmochelys imbricata*, is a marine chelonian with a circum-global distribution, but the species is critically endangered and has nearly vanished from the eastern Pacific. Although reference blood parameter intervals have been published for many chelonian species and populations, including nesting Atlantic hawksbills, no such baseline biochemical and blood gas values have been reported for wild Pacific hawksbill turtles. Blood samples were drawn from eight hawksbill turtles captured in near shore foraging locations within the Galápagos archipelago over a period of four sequential years; three of these turtles were recaptured and sampled on multiple occasions. Of the eight sea turtles sampled, five were immature and of unknown sex, and the other three were females. A portable blood analyzer was used to obtain near immediate field results for a suite of blood gas and chemistry parameters. Values affected by temperature were corrected in two ways: (i) with standard formulas and (ii) with auto-corrections made by the portable analyzer. A bench top blood chemistry analyzer was used to measure a series of biochemistry parameters from plasma. Standard laboratory haematology techniques were employed for red and white blood cell counts and to determine haematocrit manually, which was compared to the haematocrit values generated by the portable analyzer. The values reported in this study provide reference data that may be useful in comparisons among populations and in detecting changes in health status among Galápagos sea turtles. The findings might also be helpful in future efforts to demonstrate associations between specific biochemical parameters and disease or environmental disasters.

## Introduction

The hawksbill turtle, *Eretmochelys imbricata*, is a marine chelonian listed as critically endangered on the IUCN Red List (IUCN, 2015). Within the eastern Pacific, the status of hawksbills is particularly dire, with sightings so rare that populations have often been considered non-viable (Mortimer and Donnelly, 2008). The recent discovery of previously unknown nesting rookeries, along with the finding of adults in unexpected habitats, suggest that hawksbills might be somewhat more abundant than previously thought (Gaos *et al.*, 2010, 2016). Nevertheless, the species is jeopardized by low population numbers, fisheries interactions, illegal hunting, fishing gear entanglement, boat strikes, and consumption of plastics and other anthropogenic materials (Koch *et al.*, 2006; Innis *et al.*, 2010; Parra *et al.*, 2013; Denkinger *et al.*, 2013; Hardesty *et al.*, 2014; Schuyler *et al.*, 2016; Gaos *et al.*, 2016).

Although numerous studies on the ecology and behaviour of hawksbills have been carried out in the Pacific Ocean (e.g. Pérez-Castañeda *et al.*, 2007; Liles et al., 2011; Scales *et al.*, 2011; Van Houtan *et al.*, 2012) little is known about the health status of hawksbill turtles in different geographic areas. Peripheral blood chemistry and haematology parameters are useful diagnostic tools in health assessment and management (Geffre *et al.*, 2009; Zhang *et al.*, 2011; Gibbons *et al.*, 2013). To detect and monitor changes in the health of individuals and populations, it is paramount to determine species-specific normal values for blood parameters of interest. Reference intervals for juvenile Pacific hawksbills (Caliendo *et al.*, 2010), juvenile Middle East hawksbills (Hampel *et al.*, 2009) and nesting Atlantic hawksbills (Goldberg *et al.*, 2013) have been reported. Our results add to and enrich this database.

In this study, we evaluated selected blood gas, blood biochemical and haematology parameters from eight hawksbill turtles captured in the Galápagos Islands of Ecuador. The current study provides novel health assessment information on wild Pacific hawksbill turtles.

## Materials and methods

### Ethics statement

This study was performed as part of a population health assessment authorized by the Galápagos National Park Service (permit No. PC-20-14 and permit No. PC-05-15 to J.P. Muñoz-Pérez) and approved by the Universidad San Francisco de Quito ethics and animal handling protocol. All handling and sampling procedures were consistent with standard vertebrate protocols and veterinary practices.

### Study area

We studied hawksbill turtles in San Cristóbal and Española, two islands located in the southeast area of the Galápagos archipelago. Most of the captures and all recaptures occurred close to the urban area at Puerto Baquerizo Moreno in south San Cristóbal at one of two locations: Punta Carola (*n* = 2 turtles) or La Lobería (*n* = 4). In addition, one turtle was captured at Punta Pitt on the northern end of San Cristobal and one at Punta Suaréz, located in the western part of Española Island. A brief description of each location follows.

Punta Carola (0°21′26″S, 89°34′46″W) and la Lobería (0°55′40″S, 89°36′43″W) are shallow bays (1–5 m) characterized by rocky substrate although a few patches of sand also exist. Both bays are dominated by green, red and brown algae. La Lobería faces more to the south and typically has a stronger swell with cooler waters; just beyond the breaker zone depths quickly reach 100 m.

Punta Pitt (0°42′58″S, 89°14′44″W) is a shallow sandy bay (0–8 m depth) located in the northern end of San Cristóbal. The bay has a rocky reef and coral patches.

Punta Suaréz (1°22′02″S, 89°44′21″W) is located in the northwest part of Española, an island lacking human settlements. The bay is 1–18 m in depth with a sandy bottom and rocky patches.

### Turtle capture and sampling

All sampling was done between 2013 and 2016. Turtles were hand captured by swimmers using snorkel, mask and fins. To help with blood collection, captured turtles were positioned on the beach with their heads facing down the slope toward the water, a position that facilitated blood pooling in the head and neck.

After a blood sample was obtained from each turtle (see below), morphometric measurements were taken and the body temperature was measured (see below). To assist with future identification (Reisser *et al.*, 2008; Schofield *et al.*, 2008; Jean *et al.*, 2010), digital images were taken of the carapace and of each side of the head. In addition, standard, coded, metallic, Inconel self-piercing sea turtle tags (National Band and Tag Company STYLE 681IC) were affixed to the back flippers of each turtle.

### Blood sample collection and handling

Blood samples were obtained within an average of 27 min of capture. Blood samples of approximately 2.5 ml were obtained from either the left or right dorsal jugular sinus using a heparinized 1.0 or 1.5 in. 22-gauge needle attached to a 3.0 ml syringe. The blood was then immediately divided into sub-samples. Some sub-samples were used for making blood films on clean glass microscope slides, some were stored on ice in sterile plastic vials for laboratory analyses, and others were loaded into iSTAT cartridges within 10 min of sample collection. The samples stored on ice were centrifuged in the laboratory upon returning from the field on the same day and the plasma was frozen at −20°C for future analysis. The longest interval from freezing to analysis was 14 months.

### Blood gas and biochemistry parameters

An iSTAT portable clinical analyzer (Abbot Point of Care Inc., Princeton, NJ, USA) was used to obtain biochemistry, blood gas and electrolyte results using CG4+ and CG8+ cartridges (Abbot Point of Care Inc., Princeton, NJ, USA). The following parameters were measured and recorded: glucose, Hb, HCO_3_^–^, Hct, K, Na, iCa, lactate, pCO_2_, pH and pO_2_. Prior work suggests no effect of the type of iSTAT cartridge on the blood parameters and thus the values obtained from the first cartridge (CG8+) were used, except for lactate, which is only measured by the CG4+ cartridge (Lewbart *et al.*, 2014).

The iSTAT analysed the blood at 37°C then corrected pH, pO_2_ and pCO_2_ for body temperature once this information was entered. The validity of the iSTAT temperature corrections has been questioned by some authors (Chittick *et al.*, 2002; Harms *et al.*, 2003). Therefore, an independent set of corrections was calculated for pH, pO_2_, pCO_2_, following procedures previously described in detail (Lewbart *et al.*, 2014). Briefly, these corrections depended on the turtle cloacal temperature (Ti), in addition to iCa and HCO_3_^–^ values that were derived from corrected pH and pCO^2^ values, all in accordance with the equations of Kraus and Jackson (1980), Anderson *et al.* (2011a), Fogh-Anderson (1981) and Stabenau and Heming (1993).

Both sets of values (i.e. those derived from auto-corrections from the iSTAT and those derived from independent calculations) are reported. Values that were manually corrected for temperature as described above are denoted by ‘M’ subscript (Table 2).

Blood urea nitrogen (BUN), creatine kinase (CK), aspartate aminotransferase (AST), alanine aminotransferase (ALT), total protein (TP), alkaline phosphatase (ALKP), creatinine and amylase were assayed from thawed plasma using an IDEXX VetTest 8008 analyzer (IDEXX Laboratories, Westbrook, Maine 04092). Total solids were determined in five cases using a refractometer.

### Haematology

Heparinized whole blood was stored on ice immediately after collection and refrigerated. Total erythrocyte and leucocyte counts were obtained within 24 h of blood collection using Natt Herick’s stain and a Neubaeuer hemocytometer (Campbell, 1995). The blood was kept at 4°C prior to staining in the hemocytometer. Manual haematocrit was determined using high-speed centrifugation of blood-filled micro haematocrit tubes. Differential white blood cell counts were conducted by examining 100 white blood cells on a peripheral smear stained with Wright-Giemsa stain.

### Morphometric measures and body temperature

For each turtle captured morphometric measurements were taken using a flexible 100 cm measuring tape and Mini Vernier plastic Calipers (precision: 0.05 mm). These included head width (HW), curved carapace length (CCL), curved carapace width (CCW), tail to carapace length and tail to plastron length, as per standard methods. In addition, each turtle was weighed with a precision of ±0.5 kg. The sex of an immature sea turtle cannot be determined with confidence on the basis of an external examination but adults were sexed based on the external sexual dimorphism of mature hawksbill turtles (Avens and Stover, 2013). An EBRO^®^ Compact J/K/T/E thermocouple thermometer was used to obtain all temperature readings (model EW-91219-40; Cole-Parmer, Vernon Hills, IL, USA). Core body temperatures were recorded from the cloaca using the probe EBRO^®^ T PVC epoxy tip 24GA × 1 m in length, which was inserted approximately 10 cm into the cloaca.

Respiratory rates per minute were measured by visualization and the heart rate with a Doppler ultrasound probe (Parks Medical Electronics, Inc., Aloha, OR, USA) in the area of the carotid artery. Two heart rates were recorded, one as soon as possible after capture, and the second just prior to release.

### Statistical analysis

The iSTAT results for haematocrit were compared (Wilcoxon signed rank test) to the results of manually determined haematocrit. A standard alpha level of *P* = 0.05 was used for all statistical tests using R statistical software, version 3.0.2 (R Development Core Team).

## Results

### Turtle demographics and health status

The measurements and general health parameters are summarized in Table 1. For a few turtles, the body temperature, heart rate and respiration parameters were not recorded due to a lack of equipment or observers. Blood samples were drawn from a total of eight animals. Of these, three were recaptured multiple times and repeated blood samples for these individuals were obtained and analysed. The mean CCL for all turtles was 57.7 cm (SD: 14.1), with a range of 41–82 cm. The mean mass was 22.5 kg (SD: 18.6). Values ranged from 6.4 kg for the smallest immature to 60.9 kg for the largest adult female. Internal body temperature varied little among individuals, with a mean of 25.4**°**C and a range between 22.1**°**C and 27.2**°**C. All eight turtles appeared clinically healthy, had relatively little barnacle or algal growth on their carapaces, and were bright, alert and responsive.

**Table 1:** Standard measurements and vital data for wild Galápagos hawksbill turtles

Parameter	*n*	Mean	SD	Minimum	Maximum
CCL (cm)	8	56.8	14.3	41	82
CCW (cm)	8	50	12.9	34.5	71
HW (cm)	8	7.7	1.9	5.6	10
Tail-Carapace (cm)	8	7.3	1.3	5	9
Tail-Plastron (cm)	8	9.9	2.9	5.5	14
Weight (kg)	8	23.6	18.5	6.4	60.9
Body temperature (°C)	7	25	2.0	22.1	27.2
Heart1 (bpm)	6	42	7.6	36	54
Heart2 (bpm)	6	39	8.3	24	48
Respirations/min	7	3.6	1	2	5

For the three turtles captured multiple times, only the measurements obtained during the first capture are included in this table.

### Blood biochemical analysis

Tables 2–4 report the biochemistry, blood gas and haematology results. Manually determined haematocrit values were significantly different from iSTAT haematocrit values (Wilcoxon signed rank test: *W* = 1, *P* = 0.016).

**Table 2:** Mean, SD and range for blood gas and blood biochemical values for wild Galápagos hawksbill turtles

Analyte	*n*	Mean	SD	Minimum	Maximum
HCO_3_-_I_ (mmol/l)	7	33.2	5.6	23.1	39.3
HCO_3_-_M_(mmol/l)	7	32.8	5.5	22.0	39.3
TCO_2_ (mmol/l)	7	37	5	22	39
sO_2_%	7	91	2	88	95
pH_A_	7	7.264	0.084	7.147	7.401
pH_M_	7	7.275	0.072	7.180	7.382
pCO_2A_ (kPa)	7	8.1	1.3	6	9.7
pCO_2M_ (kPa)	7	8.1	1.3	6	9.7
pO_2A_ (kPa)	7	5.45	1.5	3.6	8.0
pO_2M_ (kPa)	7	10.0	1.6	8.25	12.8
Na (mmol/l)	7	157	2	154	160
K (mmol/l)	7	4.2	0.4	3.6	4.7
iCa_I_ (mmol/l)	7	1.09	0.11	0.94	1.32
iCa_M_ (mmol/l)	7	0.99	0.11	0.86	1.20
Glu (mmol/l)	7	87	10	77	105
Hct%_I_	7	30	4	24	34
Hct%_M_	8	38.9	3.6	33.3	44.0
Hb_I_(g/l)	7	96	11	82	112
Lac (mmol/l)	7	1.6	0.6	0.9	2.7
CK (μmol/dl)	7	639	309	319	1252
AST (U/l)	8	196	54	117	296
ALT (U/l)	8	38	15	18	68
TP (g/l)	8	48	7	40	58
ALKP (U/l)	8	53	26	10	86
Urea nitrogen (mmol/l)	8	18.3	7.7	6.9	29.1
Creatinine (μmol/l)	8	26	16	11	60
Amylase (μkat/l)	8	26.9	3	21.1	29.7
Total Solids (g/l)	5	43	6	36	51

iCa, ionized calcium; Glu, glucose; Hct, haematocrit; Hb, haemoglobin; Lac, lactate. ‘I’ subscript denotes values obtained through the instant iSTAT analysis, ‘M’ subscript indicates values manually corrected for temperature using standard equations and ‘A’ subscript indicates values that were auto-corrected for temperature by the iSTAT after a turtle’s cloacal temperature was entered into the iSTAT. See text for details.

**Table 3:** Mean, SD and range for manually analysed differential white cell values of wild Galápagos hawksbill turtles

Cell type	*n*	Mean	SD	Minimum	Maximum
RBC (x10^12^/l)	8	0.35	0.09	0.17	0.48
WBC (x10^9^/l)	8	5.31	3.86	1.76	12.76
Heterophil %	8	32.3	6.9	24.0	43.0
Monocyte %	8	3.6	1.9	1.5	7.5
Eosinophil %	8	18.5	4.5	10.5	24.0
Lymphocyte %	8	45.9	6.1	34.5	50.5
Basophil %	8	0.1	0.2	0.0	0.5

**Table 4:** Comparative blood values for three wild Galápagos hawksbill turtles over several time points

Turtle ID	JC268/269			JA260/261			JC236/238		
Capture dates	01/07/13	24/06/14	21/09/14	23/06/14	21/09/14	27/06/16	27/06/14	18/09/14	29/06/16
Parameters									
CCL (cm)	67.5	68	68.5	60	62	67	45	44	51.4
Body temperature (°C)	19.8	26	23.4	24.7	23.1	21.1	26	22.9	21.3
pH_M_	7.382	7.285	7.371	7.348	7.214	7.328	7.25	7.154	7.382
Na_I_ (mmol/l)	155	153	149	158	155	159	158	160	160
iCa_M_ (mmol/l)	0.88	1	0.96	1.20	1.01	1.05	1	0.96	0.81
Glu_I_ (mmol/l)	83	82	70	105	103	89	84	81	85
Hct%_M_	30	35	32	27	34	41	25	27	27
Hb_I_ (g/dl)	10.2	11.9	10.9	9.2	11.6	13.9	8.5	9.2	9.2
Lac_I_ (mmol/l)	8.35	17.9	10.5	10.4	16.7	7.2	18.3	18.9	6.5

## Discussion

Although many studies have reported baseline biochemical, blood gas and haematology values for sea turtles and other chelonian species, only a few have involved hawksbills (Hampel *et al.*, 2009; Caliendo *et al.*, 2010; Goldberg *et al.*, 2013). This study reports a set of blood values and other health parameters in wild Galápagos hawksbill turtles. Hawksbills are rare in the eastern Pacific; indeed, until recently, the species was considered essentially extinct in the region (Gaos *et al.*, 2016). The relatively small sample size (*n* = 8 plus three recaptures) represents a considerable sampling effort (56 hours in water search over a period of four sequential years).

The small sample size precluded the calculation of formal reference intervals, a process that would require a minimum of 120 individuals (Campbell, 1995; Geffre *et al.*, 2009). In addition, due to logistical constraints, all of the samples were obtained between June and September; thus, the possibility exists that sampling at different times of the year would yield somewhat different results (Lutz and Dunbar-Cooper; 1987; Casal and Orós., 2009; Fong *et al.*, 2010; Kimble and Williams, 2012).

We judged the turtles we examined to be clinically healthy and the haematology (total white cell/differential counts) and blood chemistry values were generally consistent with those reported for other healthy sea turtles (Aguirre *et al.*, 1995; Aguirre and Lutz, 2004; Flint *et al.*, 2010a, 2010b; Anderson *et al.*, 2011b; Gibbons *et al.*, 2013; Flint, 2013; Goldberg *et al.*, 2013). Minor exceptions existed, inasmuch as the pH values were slightly lower and the CO_2_ levels slightly higher than reported for loggerheads, *Caretta caretta* (Harms *et al.*, 2003), green turtles, *Chelonia mydas* (Moon *et al.*, 1997; Lewbart *et al.*, 2014) and Kemp’s ridleys, *Lepidochelys kempii* (Moon *et al.*, 1997). Another study reported higher pH values and lower CO_2_ in Kemp’s ridley turtles (Innis *et al.*, 2007). This trend of lower pH values might be due to a mild to moderate respiratory acidosis or, alternatively or additionally, be attributable to the warmer body temperatures of the Galápagos hawksbills relative to more temperate species and sampling areas. Sea turtles at cooler temperatures are known to have higher pH values (Lutz *et al.*, 1989; Moon *et al.*, 1997). It is important to note that no published blood gas values exist for wild hawksbills.

The mean hawksbill BUN value of 18.3 mmol/l is comparable to loggerheads, 23.6 mmol/l (Kelly *et al.*, 2015) and Kemp’s ridleys, 26.4 mmol/l (Carminati *et al.*, 1994), but higher than green turtles, 2.5 mmol/l (Bolten and Bjorndal, 1992) and leatherback turtles (*Dermochelys coriacea*), 1.07 mmol/l (Deem *et al.*, 2006). This might be due in part to the diet of hawksbills, which consists largely of animal protein from sponges, corals and other invertebrates (Bjorndal, 1985). Interestingly, the only other study to include hawksbill BUN values listed 7.3 mmol/l (Goldberg *et al.*, 2013), but turtles sampled in that study were nesting females. Because female hawksbills, like other marine turtles, often migrate long distances to nest in areas with limited food resources (Bjorndal, 1996; Miller *et al.*, 1998), it is likely that nesting turtles have a different nutritional status than the Galápagos hawksbills that we studied, which were captured in locations that presumably serve as foraging grounds.

The TP values in our study were higher than for juvenile hawksbills undergoing rehabilitation (Hampel *et al.*, 2009; Caliendo *et al.*, 2010) but were similar to those of nesting females (Goldberg *et al.*, 2013). This is likely due to the nutritional status of wild healthy, and reproductively sound individuals compared to younger, nutritionally challenged animals.

A number of factors are known to influence chelonian biochemistry values, including age, size and sex. For example, juvenile loggerhead turtles reportedly have lower values of albumin, calcium, globulins, haematocrit, TP and triglycerides than do adult animals, a difference probably at least partly attributable to egg production in adult females (Casale *et al.*, 2009). In green turtles, albumin, TP and triglyceride levels were found to increase with body size while calcium, glucose, potassium and sodium did not (Labrada-Martagón *et al.*, 2010). Nesting Atlantic hawksbills undergoing vitellogenesis displayed generally lower haematology and blood biochemistry values with each successive nesting event (Goldberg *et al.*, 2013). Interestingly, for the values assessed both in the Atlantic study and in our own (PCV, TP, glucose, calcium, sodium, potassium and urea), only sodium was higher in the Pacific turtles. There were some overlapping values for ALP, ALT, AST and PCV between the two populations. The small sample size in the present study, combined with the difficulty of determining the sex of immature animals in the field (Aguirre and Balazs, 2000), precluded attempts to identify possible factors contributing to variation among individuals and between populations.

A finding relevant to future methodologies is that the iSTAT proved inaccurate in determining PCV in hawksbill turtles; results were approximately 30% lower than values determined manually. A similar though smaller discrepancy has been reported in other species including loggerhead sea turtles (Wolf *et al.*, 2008), rainbow trout (Harter *et al.*, 2014), marine iguanas (Lewbart *et al.*, 2015) and Quaker parrots (Rettenmund *et al.*, 2014). Thus, manual calculation of PCV via high-speed centrifugation is recommended. Furthermore, iSTAT haemoglobin and sO_2_% values should not be considered reliable in hawksbills, because these parameters are calculated from an algorithm based on PCV.

A comparison between blood values calculated with manual corrections and with the iStat auto-corrected values suggest that the latter are usually sufficient for clinical applications in the field. Nevertheless, the findings also suggest that when accuracy is paramount, investigators studying animals with body temperatures below 37°C should be cautious about relying on auto-corrected iSTAT values for parameters that are influenced by temperature.

For the three turtles captured and sampled more than once, the CCL values for recaptures provide evidence for growth when measurements made more than one year apart are compared (Table 4). Small differences in CCL values were also recorded for sampling events just a few months apart, but these differences probably fall within the range of measurement error.

Finally, comparison of heart rates measured shortly after capture and just prior to release (an interval usually less than than 30 min) revealed that heart rate was typically lower on the second occasion (Table 1). A likely explanation is that turtles experienced a period of exertion during and immediately after capture, but recovered quickly during the period of relative inactivity that ensued.

In summary, data reported in this study represent an important step toward determining the normal range of values against which future blood gas and biochemistry results in hawksbill turtles can be compared. Such assessments are important for health monitoring and disease diagnostics. Future research should work toward establishing reference values in this species, as well as expanding sampling to facilitate comparisons of blood values across age groups and disease states.
